# Mouse Brain PSA-NCAM Levels Are Altered by Graded-Controlled Cortical Impact Injury

**DOI:** 10.1155/2012/378307

**Published:** 2012-07-15

**Authors:** Craig S. Budinich, HuaZhen Chen, Dennell Lowe, John G. Rosenberger, Joshua D. Bernstock, Joseph T. McCabe

**Affiliations:** ^1^Graduate Program in Neuroscience, F.E. Hébert School of Medicine, Uniformed Services University of the Health Sciences, 4301 Jones Bridge Road, Bethesda, MD 20814, USA; ^2^Department of Anatomy, Physiology, and Genetics, F.E. Hébert School of Medicine, Uniformed Services University of the Health Sciences, 4301 Jones Bridge Road, Bethesda, MD 20814, USA; ^3^Graduate Program in Molecular and Cell Biology, F.E. Hébert School of Medicine, Uniformed Services University of the Health Sciences, 4301 Jones Bridge Road, Bethesda, MD 20814, USA; ^4^Center for Neuroscience and Regenerative Medicine, F.E. Hébert School of Medicine, Uniformed Services University of the Health Sciences, 4301 Jones Bridge Road, Bethesda, MD 20814, USA

## Abstract

Traumatic brain injury (TBI) is a worldwide endemic that results in unacceptably high morbidity and mortality. Secondary injury processes following primary injury are composed of intricate interactions between assorted molecules that ultimately dictate the degree of longer-term neurological deficits. One comparatively unexplored molecule that may contribute to exacerbation of injury or enhancement of recovery is the posttranslationally modified polysialic acid form of neural cell adhesion molecule, PSA-NCAM. This molecule is a critical modulator of central nervous system plasticity and reorganization after injury. In this study, we used controlled cortical impact (CCI) to produce moderate or severe TBI in the mouse. Immunoblotting and immunohistochemical analysis were used to track the early (2, 24, and 48 hour) and late (1 and 3 week) time course and location of changes in the levels of PSA-NCAM after TBI. Variable and heterogeneous short- and long-term increases or decreases in expression were found. In general, alterations in PSA-NCAM levels were seen in the cerebral cortex immediately after injury, and these reductions persisted in brain regions distal to the primary injury site, especially after severe injury. This information provides a starting point to dissect the role of PSA-NCAM in TBI-related pathology and recovery.

## 1. Introduction

Traumatic brain injury (TBI) is a leading cause of death and disability throughout the world. In the United States alone, the Centers for Disease Control (CDC) report that of the nearly 1.7 million people who suffer TBI each year, over 50,000 will die [1]. A substantial number of those who survive may live with long-term TBI-related morbidities such as dementia, emotional and memory disorders, and musculoskeletal complaints [2–4]. Determining the range of outcomes following TBI is a challenge. Not only is there a remarkable degree of variability depending on the severity of injury but also establishing reliable and predictive diagnostic criteria and assessment tools used to evaluate treatment effectiveness has been formidable [5, 6]. It is obvious that TBI continues to be a significant worldwide problem demanding effective therapy that affects high yield molecular targets to decrease morbidity and mortality—a demand that has, to this point, not been met.

Studies aimed at identifying molecular targets that may improve survivability and decrease disability following TBI are manifold. However, in spite of decades of research and promising results in preclinical animal studies, no single effective therapy has successfully and consistently transferred to human clinical trials [7, 8]. It is now understood that multiple pathways converge and diverge in an intricate balance that either preserves normal function or spirals into pathology. Following the immediate and irreversible primary injury that results from the mechanical stress produced by the energy of a traumatic event, delayed and multifactorial secondary injury processes are initiated. These processes include excitotoxicity, oxidative stress, mitochondrial dysfunction, edema, inflammation, and cell death [9, 10]. Considering the heterogeneity of secondary injury processes, it can be appreciated that the levels and functional capacity of receptors and other proteins and their respective ligands and downstream signaling cascades may be adversely affected. 

One molecule that may serve as a harbinger of the complex balance between central nervous system (CNS) postinjury amelioration and exacerbation is neural cell adhesion molecule (NCAM). Described over 30 years ago, NCAM was the first cell adhesion molecule (CAM) identified [11]. NCAMs are cell surface glycoprotein members of the immunoglobulin superfamily of CAMs that are expressed in abundance in neural tissue as well as the lung, kidney, skeletal and heart muscle, and various cancers [12, 13]. Alternate splicing of at least 26 exons contained in the single NCAM gene produces three major isoforms of NCAM: NCAM-180, NCAM-140, and NCAM-120 [14]. These isoforms are categorized based upon their apparent molecular weight in kDa, and each is attached to the cell membrane via a class 1 transmembrane protein (NCAM 180 and 140) or glycosylphosphatidylinositol (GPI) anchor (NCAM 120) [15]. Regarding neural tissue expression, the general temporal and cell-type location of each isoform have been characterized. NCAM-180 expression is limited to neurons, NCAM-140 is expressed in neurons and glia, and NCAM-120 is primarily expressed in glia [16, 17]. 

Of paramount importance in maintaining the differentiation, migration, and anchoring function of NCAM during development and neurogenesis and cell survival functions in the face of insult during adulthood is the degree of expression of the posttranslationally glycosylated NCAM isoforms manifesting the large, negatively charged *α*-2,8-linked N-acetylneuraminic homopolymer named polysialic acid (PSA-NCAM). The necessity of polysialylation for efficient function of select NCAM isoforms has recently been appreciated (see Gascon et al. [18] for review). In addition to the indispensable role of PSA-NCAM in maintaining synaptic plasticity and health, recent evidence has also demonstrated the fundamental role of PSA-NCAM in preventing neurodegeneration in the face of ongoing insult such as epileptogenic central nervous system disorders [19]. Additionally, repair and recovery following TBI may be critically dependent on the generation, survival, migration, and integration of neurons from postnatal neurogenic niches into damaged circuitry in order to restore function. PSA-NCAM has been shown to be required for the survival of newly generated neurons *in vitro* [20, 21] and *in  vivo* [22]. Efficient migration of neuroblasts also appears to be reliant on PSA-NCAM expression [23]. And finally, the presence or absence of PSA-NCAM at the appropriate time following generation is believed to allow for the proper differentiation of neuronal precursors at the destined endpoint of migration [23, 24].

Considering the essential contributions of PSA-NCAM to efficient CNS function, it may be presumed that, at least in part, alterations in expression levels and function are involved in the complex pathology that follows TBI. To date, the short- and long-term alterations in expression levels of PSA-NCAM following graded-controlled cortical impact (CCI) in the mouse have not been characterized. The current study examines the immediate (two, 24, and 48 hour) and long-term (one and three week) change in the expression levels of PSA-NCAM in eight distinct regions of the mouse brain following graded-CCI that results in either moderate or severe injury. An understanding of the time course and regional distribution of PSA-NCAM alterations following TBI may contribute to the knowledge base that is being assembled to identify novel therapeutic targets.

## 2. Materials and Methods

### 2.1. Experimental Groups

All experimental procedures used in this investigation were approved by the Uniformed Services University of the Health Sciences (USUHS) Institutional Animal Care and Use Committee (IACUC). Six- to nine-week-old male C57BL/6 mice (Jackson Laboratories, Bar Harbor, ME) weighing 20–29 g were housed in an animal colony at a constant temperature (23 ± 2°C) with a 12-hour light/dark cycle and food and water *ad libitum* for at least three days prior to surgery. Mice were assigned to immunohistological (IHC) or western blot analysis groups that were sacrificed at either two, 24, or 48 hour, or one or three week postinjury time points. Either four or ten animals per injury severity group per time point were assigned for IHC or western blot analysis, respectively. Twenty additional animals were processed at 24 hours or three weeks for assessment of hemorrhage and structural alterations using whole brain and coronal section analysis. 

### 2.2. Surgery

Induction of mice was performed via spontaneous ventilation using 3% isoflurane in 100% oxygen (1.0 L per minute flow rate) for 3 minutes in a rodent volatile anesthesia box. After the application of protective ointment (Lacri-Lube) to the eyes, the head was shaved using electric clippers. Following hair removal, the head of the animal was placed in a standard stereotaxic frame and positioned using ear and incisor bars (Stoelting, Wood Dale, IL) and the skin was prepped with betadine ointment. Following skin preparation, 0.1 mL of 0.025% bupivacaine was injected subcutaneously into the planned incision site. Rectal temperature was maintained at 37°C with an isothermal heating pad and feedback controller (Stoelting, Wood Dale, IL). Anesthesia was maintained with 1.5–2% isoflurane. Unilateral CCI to the left cerebral hemisphere was performed using a modified technique previously described [25]. In brief, a 10 mm midline incision was made to expose the bregma and lambda sutures. Skin and fascia were reflected, and a 5 mm craniectomy was performed over the left parietotemporal cortex using a 0.75 mm bit attached to an electric rotary drill. A 4x microscope (Zeiss, Thornwood, NY) was used to visualize the skull during craniectomy, and intermittent saline irrigation was performed to reduce intensity of heat generated during drilling. Care was taken to avoid mechanical injury to the dura mater, and dural integrity was examined prior to impactor positioning. The impactor was positioned at a 15 degree angle to account for curvature of the skull, and the right edge of the impactor was aligned with the midline suture while the posterior edge was aligned with the horizontal portion of the lambda. The position of impact was reached after the impactor was moved 1.5 mm anteriorly and 1.2 mm to the left, thus producing a center of impact 3.0 mm anterior to the lambda and 2.7 mm left of midline. The CCI device (Impact One, Leica, Wetzlar, Germany) consisted of a computer-controlled, electromagnetically driven impactor fitted with a 3.0-mm-diameter steel tip mounted on a stereotaxic micromanipulator. The impactor tip was positioned by bringing it into contact with the exposed dura and retracted prior to setting an impact depth of 1.0 or 2.5 mm for moderate or severe injury, respectively. The goal of moderate injury was to avoid gross hemorrhage in the underlying hippocampus in an attempt to assess the effects of a possible secondary injury response on PSA-NCAM levels in regions removed from direct injury. Speed of impact was set to 5 meters/sec, and dwell time was 0.1 seconds. Following injury, the skull fragment was carefully replaced, and the incision was closed using interrupted 4.0 silk sutures. Sham mice were subjected to all of the described surgical procedures but did not receive CCI. All surgical procedures were performed in a sterile manner. All animals received a subcutaneous injection of 0.5 mL of 37°C 0.9% sodium chloride at the conclusion of the procedure to combat dehydration. Mice were placed in a heated cage to maintain body temperature until fully awake, after which they were returned to their home cage.

### 2.3. Tissue Processing

**Processing for Hemorrhage Evaluation and Tissue Disruption**

Twenty-four hours following graded-CCI, four sham, eight moderate, and four severe injury mice were administered deep anesthesia (60 mg/kg ketamine with 60 mg/kg xylazine, IP). The animals were perfused intracardially with heparinized (1,000 u/L) 0.9% sodium chloride followed by 4% paraformaldehyde in 0.1 M phosphate buffer, pH 7.4. Following decapitation, two sham, six moderate, and two severe injury brains were harvested for whole brain and coronal video microscopy (Dazor Speck Finder, St. Louis, MO, USA). Additionally, one moderate and one severe injury animal was sacrificed at three weeks. Whole brain and 1 mm coronal sections through the epicenter of the impact site were imaged to assess the extent of hemorrhage and gross tissue disruption in the brain. The remaining brains were postfixed in the skull overnight, and then removed from the skull and sequentially cryoprotected in 20 and 30% sucrose in phosphate buffer saline (PBS) until the brains sank. A frozen sliding microtome was used to acquire 30-*μ*m-thick coronal sections from the olfactory bulbs to the rostral cerebellum. Brain slices were stored in cryoprotectant [26] at −20°C until processed for assessment using standard hematoxylin and eosin (H&E) staining technique.

**Immunoblot Assessment**

Western blot analysis was performed as previously described [27, 28]. Briefly, at the time of sacrifice, each brain designated for western blot analysis was dissected on a chilled stage into eight regions (left and right cerebral cortex, hippocampus, and temporal lobe; the diencephalon and cerebellum were dissected as whole units) via a modified approach previously detailed [29]. The tissue was immediately transferred to storage in liquid nitrogen until protein extraction was performed. For protein extraction, the frozen tissue was transferred to centrifuge tubes containing ice-cold lysis buffer (50 mM Tris HCl, pH 7.4, 150 mM NaCl, 1 mM EDTA, 0.5% sodium dodecyl sulfate (SDS), 1% Nonidet P-40, 0.1% sodium deoxycholate) with complete mini protease inhibitor tablets (Roche, Indianapolis, IN, USA). Brief sonication was performed to ensure dismembranation, and the samples were tumbled at 4°C for one hour. Samples were centrifuged at 12000 rpm for 20 min at 4°C to separate the supernatant from the pellet. The supernatant was removed and protein concentrations were determined with a BCA kit (Pierce, Rockford, IL). Each sample protein concentration was normalized to either 3 or 5 *μ*g/*μ*L (determined by brain region). Equivalent amounts of protein (27–30 *μ*g) per sample were migrated through SDS-PAGE on 4–12% Bis-Tris gels (Invitrogen, Carlsbad, CA, USA). Proteins were electrophoretically transferred to a nitrocellulose membrane (Invitrogen, Carlsbad, CA, USA) that was immersed for one hour at 22°C with 5% nonfat-dried milk, and then probed with primary antibodies overnight at 4°C to evaluate levels of PSA-NCAM after CCI. An affinity-purified mouse monoclonal antibody raised against *α*-2-8 linked neuramic acid (PSA) (1 : 2000; clone 2-2B, Millipore, Billerica, MA, USA) was used. Membranes were then exposed to a donkey anti-mouse HRP-conjugated secondary antibody (1 : 5000; Vector Laboratories, Burlingame, CA) for 1.5 hours at 22°C. Detection of antibody binding was performed using enhanced chemiluminescence (ECL; Millipore, Billerica, MA, USA) and captured with the Fuji LAS-3000 imaging system (Fujifilm, Valhalla, NY, USA). To ensure equal loading of protein, membranes were stripped and reprobed with an anti-*β*-actin antibody (1 : 10000; clone AC-15, Sigma-Aldrich, St. Louis, MO, USA) and an HRP-conjugated antimouse secondary antibody (1 :  20000; Vector Laboratories, Burlingame, CA, USA). Expression of protein was quantified by normalizing the integrated density value (IDV) of the PSA-NCAM band to the IDV of the actin bands using Quantity One image analysis software (Bio-Rad, Hercules, CA).

**Immunohistochemistry Assessment**

At the designated harvest times, additional mice were euthanized (60 mg/kg ketamine with 60 mg/kg xylazine, IP) and brains were processed for immunohistochemical analysis using a modified technique previously described [30]. All animals were sacrificed in the early afternoon (1200–1500 hrs) in order to account for the rhythmic variation in PSA-NCAM level expression in the hypothalamus (see below). In brief, mice were perfused intracardially with heparinized (1,000 u/L) 0.9% sodium chloride followed by 4% paraformaldehyde in 0.1 M phosphate buffer, pH 7.4. Following decapitation, the brains were postfixed in the skull overnight, and then removed from the skull and sequentially cryoprotected in 20 and 30% sucrose in phosphate buffer saline (PBS) until the brains sank. A sliding microtome was used to acquire frozen 30-*μ*m-thick coronal sections from approximately 1.54 mm anterior to 5.02 mm posterior to the bregma. Brain slices were stored in cryoprotectant at −20°C until needed [26].

For PSA-NCAM immunohistochemical evaluation, with diaminobenzidine as the chromogen, a modified technique previously described was utilized [30, 31]. Briefly, intermittent sections spanning the above noted distance were washed in 0.01 M PBS for three 10-minute cycles on an orbital shaker to remove residual cryoprotectant. Endogenous peroxidase activity was quenched using 0.3% hydrogen peroxide in 0.01 M PBS for 30 minutes followed by a one hour incubation in 5% normal goat serum (NGS) with 0.1% Triton X-100 in PBS to block nonspecific binding sites. Sections were then incubated with antibody to PSA-NCAM (1 : 1000; clone 2-2B, mouse monoclonal, Millipore, Billerica, MA, USA) overnight at 4°C in 0.01 M PBS with 5% NGS. Following three 10 min wash cycles in 0.01M PBS the slices were incubated with biotinylated goat anti-rat or anti-mouse antibody (1 : 300; Jackson ImmunoResearch) in 2% NGS and 0.05% Triton X-100 in 0.01 M PBS for 30 minutes. Following further washes, incubation in the avidin-biotin-horseradish peroxidase (HRP) complex followed by DAB (Vectastain Elite ABC Kit; Vector Laboratories, Burlingame, CA, USA) was performed for two minutes. Sections were washed, mounted on gelatin coated slides, cleared with xylenes, and coverslipped for examination via light microscopy (Zeiss Axiovert 200, New York, NY, USA). Images were captured with a digital camera and documented using the Axiovision 4.2 software (Zeiss) package. Additionally, images were scanned and examined using the NanoZoomer microscopy apparatus and software suite (Hamamatsu, Bridgewater, NJ, USA). As a negative control, primary antibody was omitted and sections were incubated with secondary antibody and the ABC reagent prior to exposure to DAB. No immunoreactivity was noted in control tissue in which the primary antibody was withheld. Immunohistochemistry findings using DAB were evaluated by two blinded investigators. Unaltered images of the regions of interest were presented as pairs using the Microsoft Powerpoint slideshow format. Each evaluator was asked to determine which image appeared to present with the more intense degree of staining.

### 2.4. Statistical Analysis

Quantitative western blot measurements of PSA-NCAM were normalized by dividing optical density values by corresponding measures of *β*-actin derived from the same samples in each blot. All data are expressed as the mean value ± standard error (SEM) for PSA-NCAM/ *β*-actin where data across specimens were normalized to sham mean density = 1. Statistical analyses were performed using GraphPad Prism (v.5; La Jolla, CA, USA) statistical package. Injury-induced alterations in PSA-NCAM levels in immunoblot samples were compared to sham animals using a one-way ANOVA followed by Tukey's HSD test if ANOVA analysis rejected the null hypothesis. Differences were considered statistically significant at *P* < 0.05. 

## 3. Results

### 3.1. Extent of Injury

**Figure 1:**
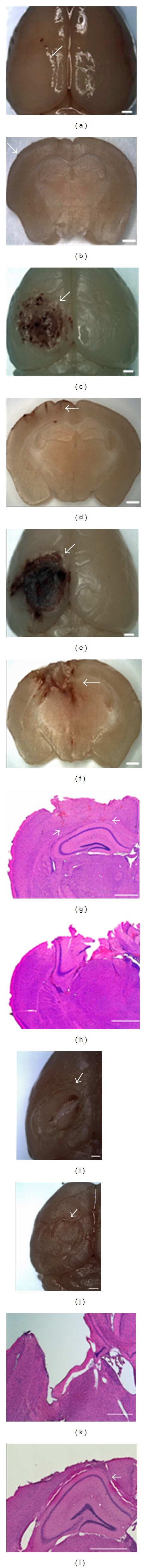
Graded injury severity. Whole brain and coronal sections displaying injury severity at 24 hours in sham (a), (b), moderate (c), (d), and severe (e), (f) injury groups. Coronal H&E sections revealed no apparent hemorrhage in the underlying hippocampus in the moderate injury animals 24 hours following injury (g). Severe injury revealed extensive hippocampal and thalamic injury at the 24 hour collection point (h). Lesions on the cerebral surface were evident three weeks following severe injury (i). Moderate injury whole brain samples displayed no apparent surface defect three weeks following injury (j). Coronal sections from brain samples obtained 3 weeks after severe CCI revealed graded substantial cortical and hippocampal damage (k) while moderate CCI resulted in loss of cortical tissue and deformity and expansion of hippocampal neuropil (l). Arrows denote region of interest. Coronal sections approximate bregma −1.70. Scale bar equals 1 mm.

Examination of tissue samples obtained 24 hours after injury revealed a progressively larger hemorrhagic lesion of the brain in the graded-CCI animals that correlated well with the depth of impact. Sham animals demonstrated no obvious injury (Figures 1(a) and 1(b)). Five of the six moderate injury (1 mm depth of impact) animals examined 24 hours after CCI had evident hemorrhagic lesions of the cortex with extension to the corpus callosum (Figures 1(c) and 1(d)). There appeared to be no apparent injury to the hippocampus, and H & E staining supported this finding (Figure 1(g)). The severe injury specimens (2.5 mm CCI depth) revealed widespread hemorrhage and structural damage to the ipsilateral cortex, hippocampus, and thalamus (Figures 1(e) and 1(f); H&E Figure 1(h)), that partially extended to the contralateral hemisphere. Evaluation of severe injury specimens obtained three weeks after CCI revealed extensive cavitation of the ipsilateral hemisphere with significant thalamic deformation (Figure 1(i); H&E Figure 1(k)). Moderate injury samples had significantly less evidence of gross anatomical distortion (Figure 1(j)); however, expansion of the dorsal ipsilateral hippocampus into the region previously occupied by the cortex was noted (Figure 1(l)).

### 3.2. Quantitative Immunoblot Findings

**Short Term Alterations in PSA-NCAM Levels after TBI**

**Figure 2:**
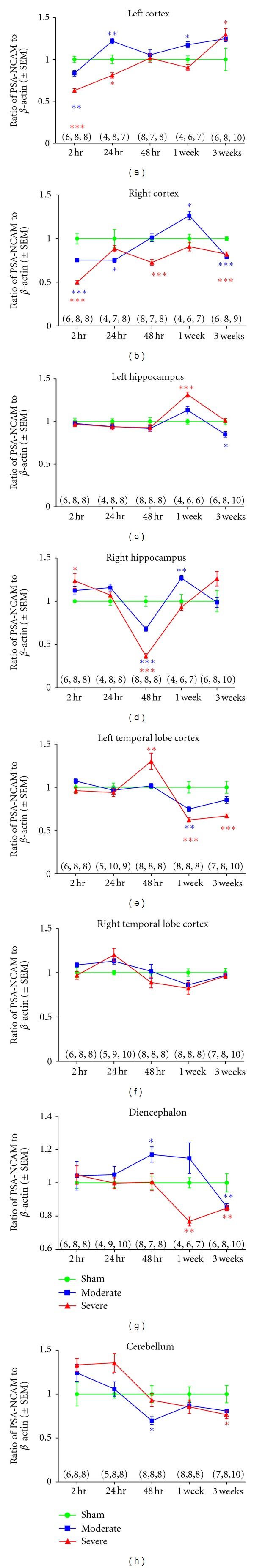
Quantitative analysis of PSA-NCAM levels following graded-CCI. Short- and long-term changes in expression levels of PSA-NCAM in the eight brain regions evaluated using western blotting. Graphs depict mean PSA-NCAM density differences ± SEM of injury groups compared to the sham group (adjusted to mean = 1). Star color indicates statistical significance of difference between injury and sham values via one-way ANOVA and *post hoc* Tukey's test (*P < 0.05, **P < 0.01, ***P < 0.001). Animal numbers per group (sham, moderate, severe) for each brain region are indicated in parentheses above the abscissa.

Western blot analysis of acute PSA-NCAM level alterations following moderate TBI revealed heterogeneity as a function of brain region (Figure 2). At 2 hours after severe CCI injury, the left cortex (the site of direct primary injury (a)) as well as the contralateral right cortex (b) exhibited decreased PSA-NCAM levels, while the other six brain regions examined did not have significant changes relative to the sham-treated group. At the 24 hour post-injury time point, the left cerebral cortex region displayed increased levels after moderate injury and reduced PSA-NCAM levels following severe CCI (a). Additionally, the right cortex responded to moderate injury with decreased PSA-NCAM levels (b) while an increase in PSA-NCAM levels in response to severe injury was seen in the cerebellum (h). A more global response was seen 48 hours following CCI. Moderate injury induced an increase in PSA-NCAM expression in the diencephalon and a decreased expression in the right hippocampus (d) and cerebellum (h). Severe injury produced a decrease in PSA-NCAM expression in the right cortex (b) and hippocampus (d) and an increase in PSA-NCAM expression in the left temporal lobe (e) 48 hrs after CCI.

**Long-Term Alterations in PSA-NCAM Levels after TBI**

**Figure 3:**
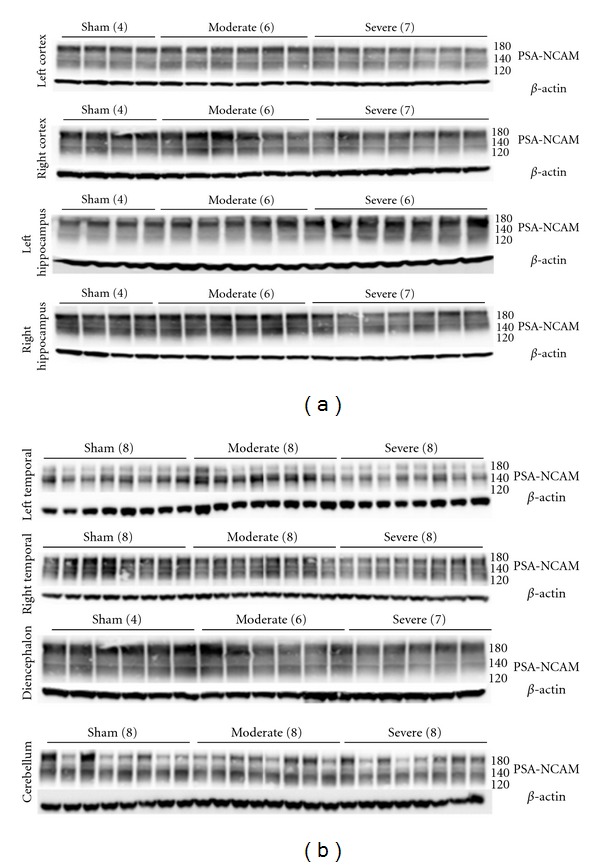
Immunoblot results for PSA-NCAM one week after CCI. One week western blot results of the eight brain regions examined following sham, moderate, or severe injury illustrate a heterogeneous response to injury. PSA-NCAM blotting is noted to span from approximately 120–250 KDa. The appearance of bands of different intensity is the result of variable degrees of polysialylation of NCAM 180, 140, and 120 (approximate weight noted on right side of image). The results represented in this figure were used to construct the one week data in Figure 2.

PSA-NCAM levels were evaluated one and three weeks after CCI (Figure 2). Seven days after CCI, PSA-NCAM changes were notable in some brain regions quite distal to the primary impact site. Levels in the left hippocampus (c), underlying the impact site, were elevated while more distal brain regions (the left temporal lobe (e), diencephalon, and cerebellum) exhibited significantly reduced levels; particularly after severe CCI. Western blots for one week samples are presented in Figure 3. Reduced levels persisted in the left temporal lobe (e), three weeks after injury, and were still evident in the diencephalon (g); even after moderate CCI. Severe injury resulted in decreased PSA-NCAM expression in the cerebellum at three weeks.

### 3.3. Qualitative Immunohistochemistry Findings

**Figure 4:**
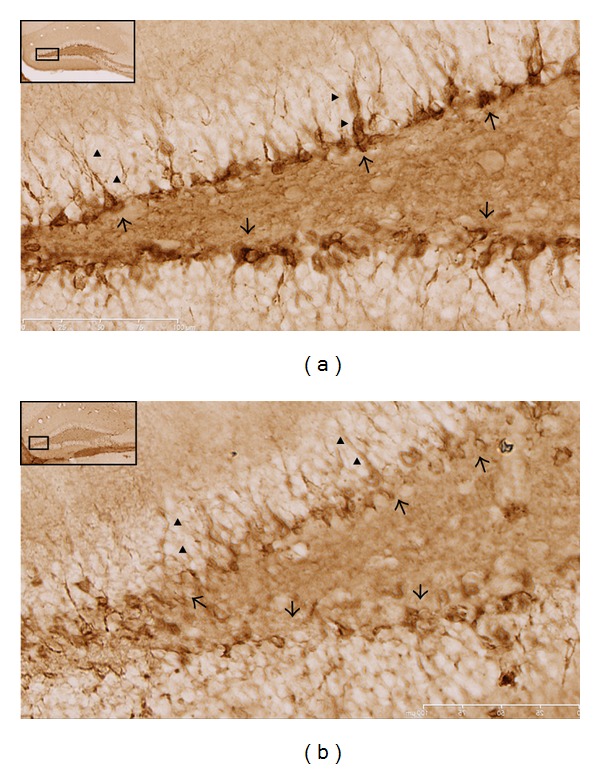
Comparison of PSA-NCAM levels in right dentate gyrus of sham and severe injury animals 48 hours following surgery. PSA-NCAM staining of subgranular zone cells in the contralateral hippocampus reveals more intense expression in the soma (arrows) and processes (arrow heads) of sham (a) versus severe (b) injury animals. Images are 40(x) magnification; scale bar = 100 *μ*m.

**Figure 5:**
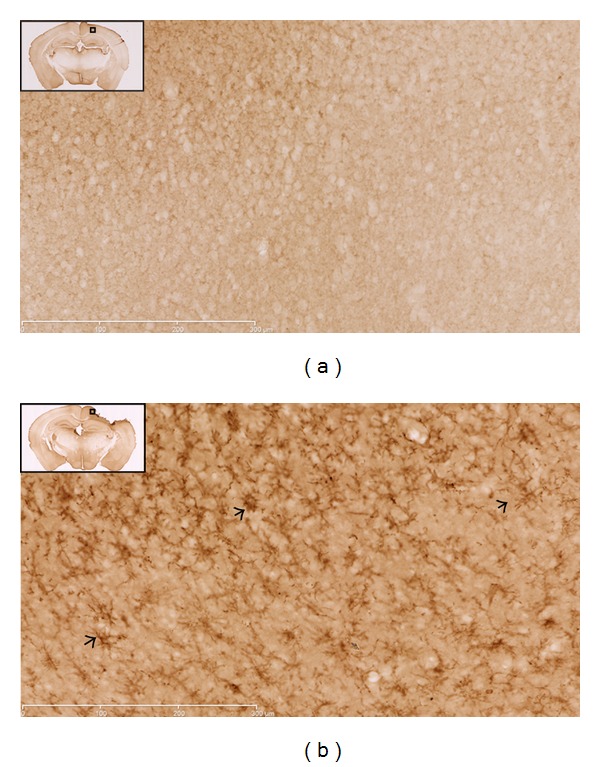
Immunohistochemical evidence of PSA-NCAM expression alterations in the left cortex one week following injury. (a) Sham animals demonstrate subtle, uniform PSA-NCAM staining in the left cortex one week following craniectomy. (b) PSA-NCAM expression in the ipsilateral cortex of moderate injury animals is significantly increased with particularly dark staining of cells that display the appearance of reactive astrocytes (arrows). Images are 20(x) magnification; scale bar = 300 *μ*m.

**Figure 6:**
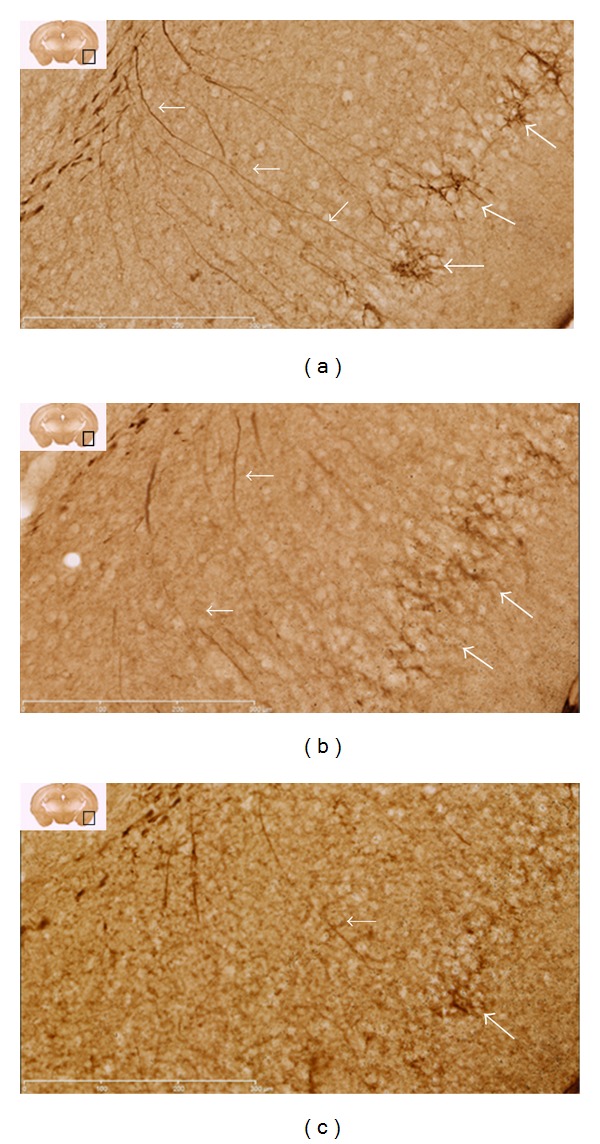
PSA-NCAM staining in left temporal lobe one-week following graded-CCI. Diaminobenzidine staining for PSA-NCAM in sham (a), moderate (b), and severe (c) injury animals one week after CCI demonstrates decreased staining of the soma (large arrows) and apical dendrites (small arrows) of neurons in the piriform cortex. Insert denotes approximate region of interest at approximately bregma −1.70 mm. Images are 20(x) magnification; scale bar = 300 *μ*m.

Qualitative evaluation of tissue slices was undertaken to further examine PSA-NCAM changes seen with western blotting. The approach was to carefully survey all regions of brain sections that were obtained at the indicated time points after injury that we believed would produce large enough alterations in expression as to be detected using microscopy. In spite of attempts to process tissue sections identically, variability within groups was observed, so that qualitative findings intermittently supported the trends revealed with immunoblotting. For example, the two hour samples from immunoblot analysis indicated that PSA-NCAM expression decreased in the right cortex of both injury groups at this time point, but a noticeable difference between groups was not reliably observed using immunohistochemical evaluation (data not shown). Likewise, PSA-NCAM expression in the medial prefrontal cortex did not appear to be different between groups. These observations are partially anticipated as the general PSA-NCAM expression pattern in most of the cortex does not show a distinct structural distribution (with exceptions; see below for temporal lobe PSA-NCAM staining) and appears as faint labeling of neuropil or immunonegative [32]. In agreement with immunoblot findings, assessment of right sham and severe injury hippocampi revealed a distinct decrease in PSA-NCAM staining of the soma and processes of cells located in the subgranular zone of the dentate gyrus of severely injured animals (Figure 4). Assessment of the left cortex at one week confirmed the increased PSA-NCAM expression levels seen in moderate injury animals (Figure 5). Of note, immunohistochemical evaluation revealed that expression of PSA-NCAM on cells displaying morphological characteristics of reactive astrocytes accounts for a significant proportion of the increase. Additionally, evaluation of the left temporal lobe one week following graded-CCI also confirmed immunoblot data; an obvious decrease of PSA-NCAM labeling of the soma and apical dendrites (Figure 6) of what are most likely layer II semilunar cells of the olfactory cortex [33].

## 4. Discussion

### 4.1. Alterations in PSA-NCAM after TBI

Originally thought to be only expressed in the CNS during embryonic development [34], PSA-NCAM manifestation in distinct regions of the adult CNS illustrates an important lifelong role of the molecule in maintaining nervous system plasticity. Of note, PSA-NCAM is constitutively expressed in some brain regions related to neuroendocrine and behavioral functions [35, 36]. Recent findings also indicate that NCAM function can be dramatically altered by multiple factors, and that the biosynthesis of PSA-NCAM is intimately related to increases in cell axon activity and alterations in cell input [37].

The qualitative and quantitative distribution of PSA-NCAM in the uninjured adult CNS has been examined and characterized in previous studies [32], but no reports describe the fate of PSA-NCAM in the mechanically contused brain. The results of the present study show that graded-CCI induces short- and long-term increases and decreases in PSA-NCAM levels in a range of brain areas. Furthermore, the alterations in these levels were heterogeneous and complex events over time with distinct patterns of change across anatomically separated brain regions. These alterations may have important implications for understanding neural plasticity changes and repair responses following brain injury, including proximal and distal interactions between various brain regions as a function of injury severity.

Following injury in the parietal cortex, levels exhibited fluctuations that included an initial increase in PSA-NCAM levels in the region of primary impact in the left parietal cortex in moderately-injured animals, while levels were depressed when the injury was severe. Correspondingly, in the contralateral hemisphere, PSA-NCAM reductions in the corresponding parietal cortex remained for a longer time after moderate injury and were persistently depressed after severe CCI. Perhaps the final outcome following CCI is telling. At three weeks after CCI, levels of PSA-NCAM were elevated in the left parietal lobe that sustained primary injury, while levels were reduced in the right parietal cortex and the diencephalon. In the left temporal lobe, levels were not reduced during the initial two days after injury, but then were depressed by the first and third week post-injury. However, in the contralateral temporal lobe, PSA-NCAM levels were unaffected by the injury. These data may belie the overall direct versus indirect connections of the injured parietal cortex with proximal and more remote structures. Specifically, by three weeks after injury there was a “local” increase in PSA-NCAM levels as a result of reorganization at the primary site of injury, while regions with more direct and robust connections to the injured site (the thalamus, the contralateral parietal region, and the ipsilateral temporal lobe) exhibited diminished levels. Conversely, the more distal, contralateral temporal lobe appears to have been unaffected in terms of PSA-NCAM levels. Although we were unable to fully corroborate the PSA-NCAM changes seen in the hippocampus via western blotting efforts with the results obtained using IHC, these alterations too may have implications in long term recovery. For example, the immunoblot data indicated an increase in PSA-NCAM levels at one week in either the ipsilateral or contralateral hippocampi following severe or moderate TBI, respectively. Even though variability in the intensity of staining prevented us from isolating the location and degree of these changes, these alterations may indicate the response of a group of immature neurons in the subgranular zone reacting to injury [39]. This increase in PSA may promote efficient migration of these neurons to the site of injury and reintegration into hippocampal circuits [40]. Additionally, the increased PSA expression may also be occurring on reactive astrocytes as a component of the formation of a glial scar.

While the present results are one of the first to examine PSA-NCAM alterations after controlled cortical impact, the role of PSA-NCAM in prosurvival pathways initiated during CNS excitotoxicity insult has been investigated [19]. In this study, endoneuraminidase (endoN) removal of PSA from NCAM following hippocampal injection of kainic acid (KA) in mice was used to determine that PSA-NCAM serves as an indispensable coreceptor of GDNF family receptor *α*1 (GFR*α*1) to mediate prosurvival Ret-independent glial cell-derived neurotrophic factor (GDNF) initiated Fyn-FAK-MAP kinase pathway activation. It was determined that retrograde neurodegeneration was reduced in the presence of PSA-NCAM in the contralateral hippocampus, thus demonstrating the ability of PSA-NCAM to affect survival in the KA-treated hippocampus via GDNF signaling across the dorsal hippocampal commissure [19]. Additionally, an interesting neuroprotective relationship between heat shock protein 70 (HSP-70) and PSA-NCAM has been revealed [39, 41]. PSA-NCAM has also been shown to protect hippocampal neurons from glutamate-induced excitotoxic death most likely by inhibition of NR2B subunit-containing NMDA receptors [42]. These investigations serve as evidence supporting the importance of PSA-NCAM on the cell survival response following injury. Post-injury alterations in PSA-NCAM expression demonstrated in our study makes it difficult to confidently explain the significance of an alteration in an isolated brain region at a specific time point. However, an early decrease in PSA-NCAM expression may perpetuate a feed-forward injury cycle that could amplify long-term neuropathology. Conversely, the increased PSA-NCAM at the primary injury site noted in our study at one and three weeks may reflect an attempt to enhance progenitor differentiation and neuronal survival or act as a compensatory mechanism in response to probable TBI-induced BDNF dysfunction [43–45]. 

### 4.2. Therapeutic Potential

**Figure 7:**
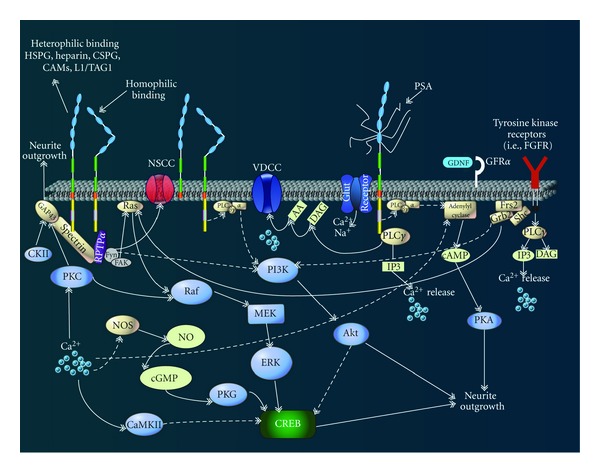
NCAM and PSA-NCAM signaling pathways. Currently accepted signaling pathways believed to be involved in neurite outgrowth; the most studied and understood result of NCAM activation. NCAM-180 and- 140 are represented by five Ig like domains and two fibronectin III domains extracellularly and an intracellular segment of varying length. NCAM-120 is attached to the membrane via a GPI anchor. Color designations: tyrosine kinases = red; other protein kinases = blue; nonproteins = silver. Abbreviations: AA, arachidonic acid; cAMP, cyclic adenosine monophosphate; CREB, cAMP response element-binding protein; CaMK, Ca^2+^-calmodulin-dependent kinase; cGMP, cyclic guanosine monophosphate; CKII, casein kinase II; DAG, diacylglycerol; ERK, extracellular regulated kinase; FAK, focal adhesion kinase; FGFR, fibroblast growth factor receptor; Frs2, FGFR substrate; GAP-43, growth-associated protein 43; NO, nitric oxide; NOS, NO synthase; NSCC, nonspecific cation channel; PI3K, phosphatidylinositol 3-kinase; PKA, protein kinase A; PKC, protein kinase C; PKG, protein kinase G; PLC, phospholipase C; RPTP, receptor protein tyrosine phosphatase; VDCC, voltage-dependent Ca^2+^-channel. Broken lines indicate putative intracellular interactions. Illustration adapted with permission from [18, 38].

PSA-NCAM's involvement in cell signaling is both far reaching and essential for proper nervous system function.  Figure 7 presents an overview of the currently understood signaling pathways initiated by NCAM and PSA-NCAM. Neurotrophin activity (i.e., brain-derived neurotrophic factor (BDNF)) ensuring appropriate neuron differentiation, plasticity, and ultimately survival or death appears to require PSA expression [18]. The interaction of PSA-NCAM with tyrosine kinase (Trk) and tumor necrosis factor (TNF) receptors is the most likely mechanism of action [46, 47]. Broadly speaking, the interaction between PSA-NCAM and various cell adhesion molecules, proteoglycans, and cell surface receptors allows it to serve as a modulator of cell-cell and cell-extracellular matrix interactions in order to allow for preservation and migration of neuronal and glial progenitors, modulation of synaptic plasticity, and the refinement of survival and maintenance roles throughout the cell life cycle [48].

Do the alterations in PSA-NCAM expression following TBI present as possible targets of therapy? The ability of PSA to modulate cell interactions in order to promote plasticity, precursor cell migration, and axonal defasciculation and targeting, raises the possibility of using PSA gain-of-function to augment the regenerative response of the brain to injury. Our study reveals several potential therapeutic treatment windows for PSA therapy to promote function of endogenous recovery mechanisms while combating the postinjury inhibitory environment in the brain. For example, PSA-NCAM expression was significantly decreased three weeks following injury in multiple brain regions including the cerebral and temporal cortices, hippocampus, diencephalon, and cerebellum following moderate or severe injury. In some cases, this decrease followed an earlier period of increased expression of PSA-NCAM (Figure 2). Multiple studies reinforce the idea of overexpressing PSA in astrocytes or Schwann cells to produce a permissive environment for axonal regeneration following brain injury. For example, to maintain and increase PSA expression in the cerebellum of L1/GAP-43 transgenic mice, a lentiviral vector carrying polysialyltransferase (ST8SiaIV) cDNA was injected into a cerebellar stab wound or used to transfect Schwann cells that were subsequently transplanted into the site of injury [49]. As a result of these interventions, Purkinje cell axonal sprouting was enhanced, and the density of sprouting in the PSA-enhanced Schwann cell graft was nine-times greater than that seen in non-PSA vector transfected grafts at two months. The *overexpression* of PSA appears to be critical to this process because in the cerebellum of wild-type mice PSA is present, and late axonal sprouting occurs, at the injury site 12 months after axotomy [50]. Perhaps the antiadhesive properties of an immediate increase in PSA expression in the wound following injury could combat the inhibitory effect of chondroitin sulfate proteoglycans (CSPGs) that are expressed rapidly following injury [51]. **In * vitro* and *in vivo* studies have noted that ectopic expression of PSA in Schwann cells has been shown to improve initial post-injury migration and enhance earlier and more effective remyelination when compared to endogenous Schwann cells [52, 53]. Because PSA seems to be a permissive molecule that does not override normal cell interactions, it does not appear to have a deleterious effect on uninjured tissue function [54]. Additionally, increased PSA expression in the cortex and hippocampus could enhance the migration of progenitors from the SVZ and SGZ to the site of injury [55]. All of the above evidence provides an encouraging foundation to begin exploring NCAM and PSA-NCAM CNS repair efforts with such efforts as mimetic compounds and molecules that alter biosynthesis of these versatile and potentially healing adhesion molecules.

### 4.3. Summary

Traumatic brain injury is a devastating and life altering event that is sharply increasing in incidence throughout the world, particularly among military members, as well as in civilian populations in developing countries where there is a recent increase in motor vehicle use [56]. An ever expanding understanding of the mechanisms responsible for the acute and chronic pathology following TBI has allowed for epidemiological associations to be made between TBI and the development of chronic neurodegenerative disease. In particular, an appreciation of the complex workings of the secondary injury cascade has led to the exploration of the role of several molecules as contributors to pathological processes or potential therapeutic targets. A particular class of adhesion molecule that has received little attention regarding a potential role in TBI is PSA-NCAM.

In this study we examined changes in PSA-NCAM expression following graded-CCI in the mouse. Severe and moderate injury produced immediate as well as long-term alterations in PSA-NCAM expression both proximal and distal to the impact site. Alterations in these species of adhesion molecule have been shown to result in acute and long-lasting alterations in neuron migration, neurite formation and axon fasciculation, synapse development and function, memory function, and emotional status. The significant expression level changes seen in our study may contribute to dysfunction and/or healing following injury.
